# Codon Bias of the *DDR1* Gene and Transcription Factor EHF in Multiple Species

**DOI:** 10.3390/ijms251910696

**Published:** 2024-10-04

**Authors:** Zhiyong Zhang, Wenxi Li, Ziyang Wang, Shuya Ma, Fangyuan Zheng, Hongyu Liu, Xiaodong Zhang, Yueyun Ding, Zongjun Yin, Xianrui Zheng

**Affiliations:** College of Animal Science and Technology, Anhui Agricultural University, Hefei 230036, China; zzy_314864016@163.com (Z.Z.); 15543601529@163.com (W.L.); wangziyang@stu.ahau.edu.cn (Z.W.); 15136006190@163.com (S.M.); 23721395@ahau.edu.cn (F.Z.); liuhongyu@ahau.edu.cn (H.L.); xdzhang1983@ahau.edu.cn (X.Z.); dingyueyun@ahau.edu.cn (Y.D.)

**Keywords:** *DDR1*, EHF, codon usage bias, cattle, lactation regulation

## Abstract

Milk production is an essential economic trait in cattle, and understanding the genetic regulation of this trait can enhance breeding strategies. The discoidin domain receptor 1 (*DDR1*) gene has been identified as a key candidate gene that influences milk production, and ETS homologous factor (EHF) is recognized as a critical transcription factor that regulates *DDR1* expression. Codon usage bias, which affects gene expression and protein function, has not been fully explored in cattle. This study aims to examine the codon usage bias of *DDR1* and EHF transcription factors to understand their roles in dairy production traits. Data from 24 species revealed that both *DDR1* and EHF predominantly used G/C-ending codons, with the GC3 content averaging 75.49% for *DDR1* and 61.72% for EHF. Synonymous codon usage analysis identified high-frequency codons for both *DDR1* and EHF, with 17 codons common to both genes. Correlation analysis indicated a negative relationship between the effective number of codons and codon adaptation index for both *DDR1* and EHF. Phylogenetic and clustering analyses revealed similar codon usage patterns among closely related species. These findings suggest that EHF plays a crucial role in regulating *DDR1* expression, offering new insights into genetically regulating milk production in cattle.

## 1. Introduction

Milk production is regulated by various genes and factors. Our previous ATAC-seq and RNA-seq analyses of mammary gland tissues at different lactation stages in cows revealed that the discoidin domain receptor 1 (*DDR1*) gene is a promising candidate gene that influences dairy production traits. Furthermore, the ETS homologous factor (EHF) transcription factor (TF) was predicted to regulate the expression of *DDR1* based on the animal transcription factor database (TFDB) 4.0. Moreover, *DDR1* has been shown to play a pivotal role in various dairy production traits [1,2,3,4]. The *DDR1* gene is a member of the receptor tyrosine kinase family (PTK), and its ligand is collagen [5,6]. Recent studies have indicated that aberrant activation of the *DDR1* gene is a key factor in the initiation and progression of tumors [7,8]. Further investigation into *DDR1* revealed a connection with the breast. Initial studies in mice have demonstrated that the mRNA level of *DDR1* increases during pregnancy [2]. Subsequent studies employing mice, in which the *DDR1* gene had been ablated, have indicated that *DDR1* regulates the proliferation of mammary epithelial cells within the mammary glands [9]. These findings suggest that *DDR1* plays a pivotal role in mediating the extracellular matrix in the mammary gland. Subsequently, research conducted on mouse HC11 mammary epithelial cells has indicated that lactation might be caused by the prolactin pathway and the *DDR1* gene working with the extracellular matrix pathway. This has been demonstrated by the overexpression of collagen and the *DDR1* gene, both of which activate Stat5 and increase β-casein [3].

An analysis of common expression networks and miRNA pathways that affect milk yield in dairy cows has identified *DDR1* and *DDHX1* as key genes in the miRNA regulatory network that regulates milk yield [1]. Furthermore, the *DDR1* gene and ErbB2/ErbB3 proteins have been shown to regulate the expression in mammary epithelial cells through a regulatory pathway [10]. Given this evidence, it can be reasonably concluded that the *DDR1* gene controls the growth and metabolism of mammary epithelial cells, as well as the development of mammary glands in mammals, by regulating downstream primers, such as the extracellular matrix, Stat5, ErbB2/ErbB3, and other factors. TFs are proteins that possess DNA-binding domains (DBDs) that enable them to recognize specific DNA sequences and, subsequently, control the expression of genes in all organisms [11]. Therefore, it has been postulated that *DDR1* regulates lactating cells in dairy cows, a phenomenon which affects the development of the mammary gland, milk synthesis and secretion, and the growth and metabolism of mammary epithelial cells [1,9,12]. In addition, this study provides a theoretical foundation for enhancing milk quality and increasing milk production. TF plays pivotal roles in the regulation of gene expression, driving species evolution, influencing animal genetics, and affecting diseases. Moreover, the evolution of gene regulation and species diversity is facilitated by TF regulatory networks and target prediction [13,14,15,16,17]. Based on an analysis of the available literature and data provided by the Animal TFDB 4.0, we have identified TFs that are potentially involved in the regulation of *DDR1*. The results have revealed that EHF may play a role in lactation. EHF, also known as epithelial-specific ESE-3, is a notable member of the ETS superfamily, which comprises the ESE subfamily [18]. Research indicates that the level of EHF activity varies depending on the specific type of cancer. For example, some malignancies benefit from TFs, such as gastric and ovarian cancers [19,20], whereas oral and colon cancers [21,22] use TFs to limit tumor growth. EHF affects breast epithelial cells and plays a significant role in the development of breast cancer [23]. A notable increase in EHF has been observed by using qPCR in a mouse model of transplanted breast cancer [24]. Further research has indicated that the EHF TF expression is significantly higher in breast cancer cells than in normal epithelial cells [25]. The present hypothesis posits that the EHF TF influences the development of the mammary gland and, subsequently, milk productivity. This hypothesis is based on an analysis of existing research on these factors.

Transcription is the process by which genetic information is transferred from DNA to mRNA, with codons formed at intervals of three nucleotides. Except for five special codons, the remaining 18 amino acids are encoded by multiple synonymous codons, a phenomenon known as codon parsimony [26,27]. Codon usage bias (CUB) refers to the unequal utilization of synonymous codons encoding distinct amino acids, and it shows that certain codons are employed at a higher frequency than others [28,29,30]. The evolution of the genome has been influenced by CUB, which can be attributed to a combination of genetic mutations, natural selection, and genetic drift [31,32,33]. The following factors influence CUB: CG heterogeneity [34], tRNA abundance [35], gene length [36], mRNA secondary structure [37], protein hydrophobicity [38], and amino acid conservation. Understanding codons has also facilitated the gradual evolution of relevant codon preference research methods. The current method of analyzing codon characteristics employs the following parameters: codon adaptation index (CAI) [39], effective number of codons (ENC) [40], frequency of optimal codons (FOC) [41], and GC3. Moreover, principal component analysis (PCA) and cluster analysis have recently garnered significant attention in the field of codon preference analysis [42]. Given the contributions of diverse approaches and viewpoints to the establishment of codon preference analysis methods, it is essential to recognize these distinctions and their respective foci when analyzing methods and conducting joint studies. Studies on codon preference have enabled the acquisition of detailed information regarding gene sequences that may be employed to make well-informed decisions regarding species selection and improvement; moreover, such data may exert a considerable influence on the evolution of genomes [43]. Codon bias research facilitates the analysis of several important areas within the field of biological evolution and phylogeny, including the molecular evolution of genes, horizontal gene transfer between species, protein-coding regions, and DNA translation. In addition, it may influence the regulation of genes over time and cycles, the structure and function of proteins, and the expression of high-level genes [28]. Increased expression of recombinant or insect-resistant proteins in plants has been achieved by codon preference. Bollworm resistance in transgenic crops is caused by the significant expression and synthesis of Bt proteins in cotton via codon preference [44]. Codon bias represents a viable approach for bioremediation, as evidenced by its capacity to enhance P450 protein synthesis in monocotyledons through heterologous systems [45]. Moreover, gene expression and protein shape of *Pichia pastoris* in a yeast expression system has been regulated through the use of codon bias [46]. The study of codon preference facilitates the extraction of distinctive data regarding gene sequences, which may exert a significant influence on genome evolution and serve as a theoretical foundation for species improvement and selection [43].

This study analyzes the CUB of the *DDR1* gene and the corresponding CDS region of the EHF TF across 24 species and explores the correlations and phylogenetic relationships between the CUB of *DDR1* and EHF TF. This research offers new insights into the codon and phylogenetic relationship between the EHF TF and *DDR1* and may provide a novel theoretical basis for enhancing milk production performance in cattle.

## 2. Results

### 2.1. Construction of DDR1 Gene and EHF Phylogenetic Trees

**Figure 1:**
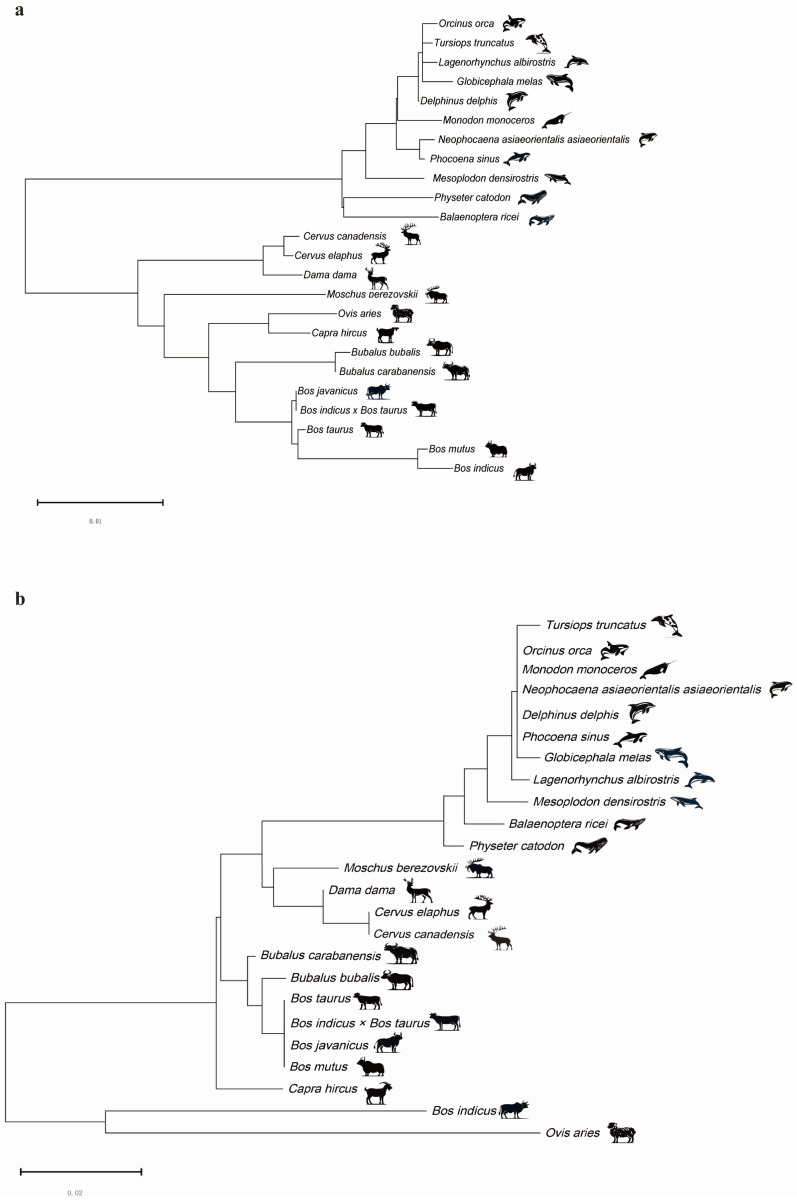
Construction of *DDR1* gene and EHF phylogenetic trees. (**a**) Phylogenetic tree constructed based on the CDS sequence of the *DDR1* gene. (**b**) Phylogenetic tree constructed based on the CDS sequence of the EHF TFs.

To examine similarities in species relatedness, phylogenetic trees were constructed for both *DDR1* and EHF. The phylogenetic tree for *DDR1* (Figure 1a) revealed that aquatic and terrestrial animals were clustered together. The phylogenetic tree for EHF (Figure 1b) yielded comparable results, with the species in the same branch displaying a broader range of relatedness. In addition, there was evidence of close kinship among species, as the *DDR1* gene node and TFs of EHF were found to be shared by *Tursiops truncatus*, *Orcinus orca*, *Globicephala melas*, *Bos indicus* × *Bos taurus* cross, and *Bos javanicus*. Moreover, *Bos taurus*, *Bos mutus*, *Physeter catodon*, and *Balaenoptera ricei* were projected to connect to a higher-level node on one side and share a node with each other, thereby demonstrating comparable affinities. These findings suggest that *DDR1* and EHF are comparable in their capacity to predict species affinities.

### 2.2. Codon Usage Patterns for the DDR1 Gene and EHF

To investigate CUB and adaptations in the coding sequences (CDS) of EHF and *DDR1*, we calculated 14 contributor correlation indices using CodonW 1.4.4 [47]. The usage frequencies of C3s and G3s in *DDR1* were higher than those of A3s and T3s, with a mean of 75.49% for GC3s which is significantly greater than the random distribution of 50% (Table 1). Similarly, in EHF, the frequencies of C3s and G3s also surpassed those of A3s and T3s, with a mean of 61.72% for GC3s which is also above the random distribution (Table 2). A comparison of the main contributor values for *DDR1* and EHF indicated a preference for G/C in both, although codons ending in G/C were favored in *DDR1*. ENC and CAI, as important metrics for codon analysis, showed similar preferences in *DDR1* and EHF (Table S1). These results suggest that codons exhibit comparable preferences in *DDR1* and EHF, with *DDR1* showing a stronger inclination toward G/C endings.

### 2.3. Relative Synonymous Codon Usage (RSCU) Values Analysis and Determination of Putative Optimal Codons for DDR1 and EHF across 24 Species

To gain insights into CUB and optimal codons of *DDR1* and EHF, we conducted statistical analyses. The results demonstrate the presence of 23 codons with a RSCU value greater than 1 in *DDR1* (Table 3). The codons identified were UUC, CUC, CUG, AUC, GUG, UCC, CCC, ACC, GCC, UAC, CAC, CAG, AAC, AAG, GAC, GAG, UGC, CGC, CGG, AGC, AGG, GGC, and GGG. All 23 codons terminated in G/C. In contrast, 24 codons with RSCU values greater than 1 were identified in EHF: UUC, CUC, CUG, AUC, GUC, GUA, GUG, UCC, CCU, ACC, GCC, UAC, CAC, CAG, AAC, AAA, GAC, GAA, UGC, CGA, CGG, AGC, AGA, and GGG (Table 4). Among the codons of *DDR1* and EHF, six codons ended with A/U, whereas the remaining 18 codons predominantly ended with G/C. This further reinforces the observed preference for G/C-ending codons for both *DDR1* and EHF TFs. Analysis of the *DDR1* and EHF codons revealed that 27 low-frequency codons (RSCU < 1) and 17 high-frequency codons (RSCU > 1) were shared between the two (see Figures S1 and S2).

The optimal codon was identified as AGG, which exhibited ΔRSCU ≥ 0.08, high > 1, and low < 1 based on the RSCU values of the *DDR1* gene across 24 species (Table S2). The optimal codons for EHF were CCC, UAC, and GGA (ΔRSCU ≥ 0.08, high > 1, and low < 1) (Table S3).

### 2.4. Hierarchical Clustering Analysis of RSCU for DDR1 and EHF

**Figure 2:**
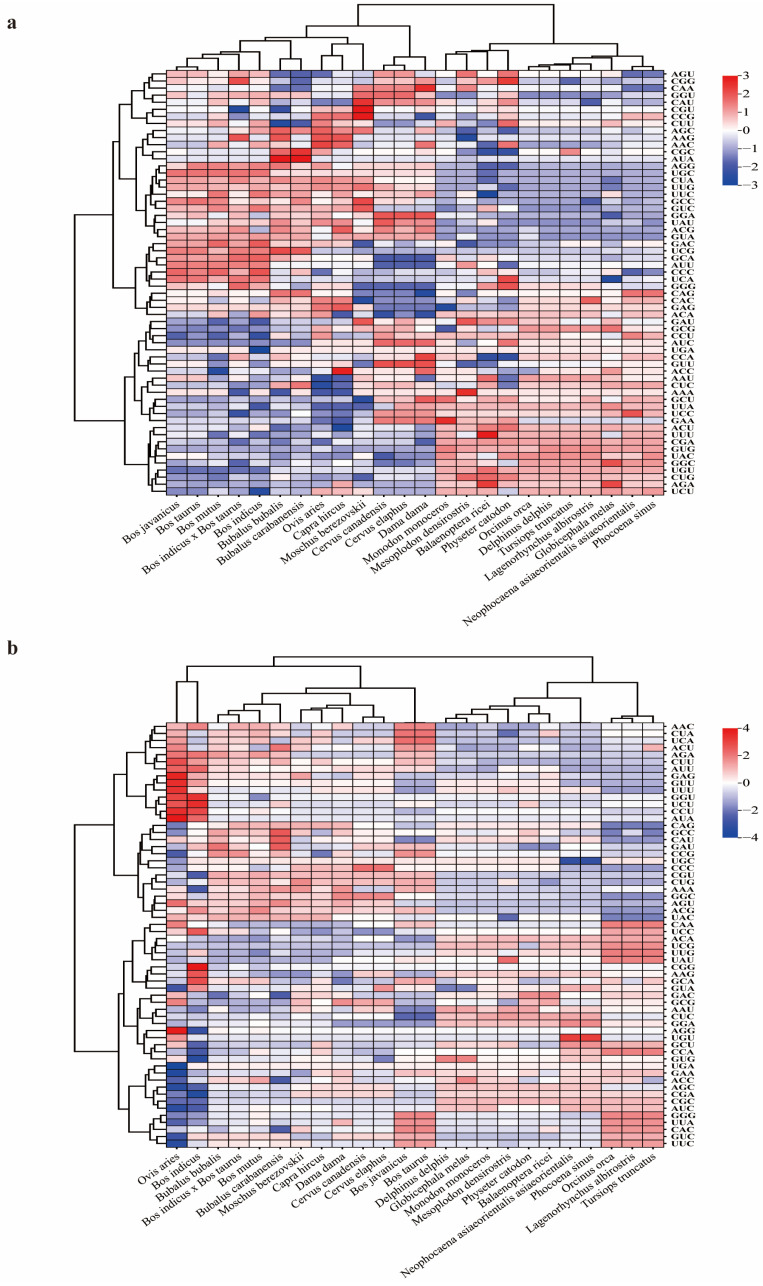
Hierarchical cluster analysis. (**a**) Cluster analysis of 59 codon systems in the CDS region of the *DDR1* gene among 24 species. (**b**) Cluster analysis of 59 codon systems in the CDS region of the EHF TFs among 24 species.

Hierarchical clustering analysis of the RSCU values of *DDR1* and EHF (Figure 2a,b) revealed that aquatic and terrestrial animals were grouped separately, an output which aligns with the findings from the phylogenetic tree. Furthermore, species that demonstrated close affinities for both the *DDR1* gene and EHF TFs, such as *Bos javanicus* and *Bos mutus*, also exhibited a similar pattern in the hierarchical clustering analysis. Moreover, the results of the hierarchical cluster analysis were in accordance with the predicted affinities indicated by the phylogenetic trees, as exemplified by *Tursiops truncatus*, *Orcinus orca*, and *Lagenorhynchus albirostris*. These findings suggest that the CDS of the *DDR1* gene and EHF TF, along with their associated codons, display similarities in species affinities. Furthermore, identical codon RSCUs were identified in the CDS sequences of *DDR1* and EHF in the species with comparable affinities.

### 2.5. PCA Analysis of 24 Species and Codons Separately Using RSCU Values

**Figure 3:**
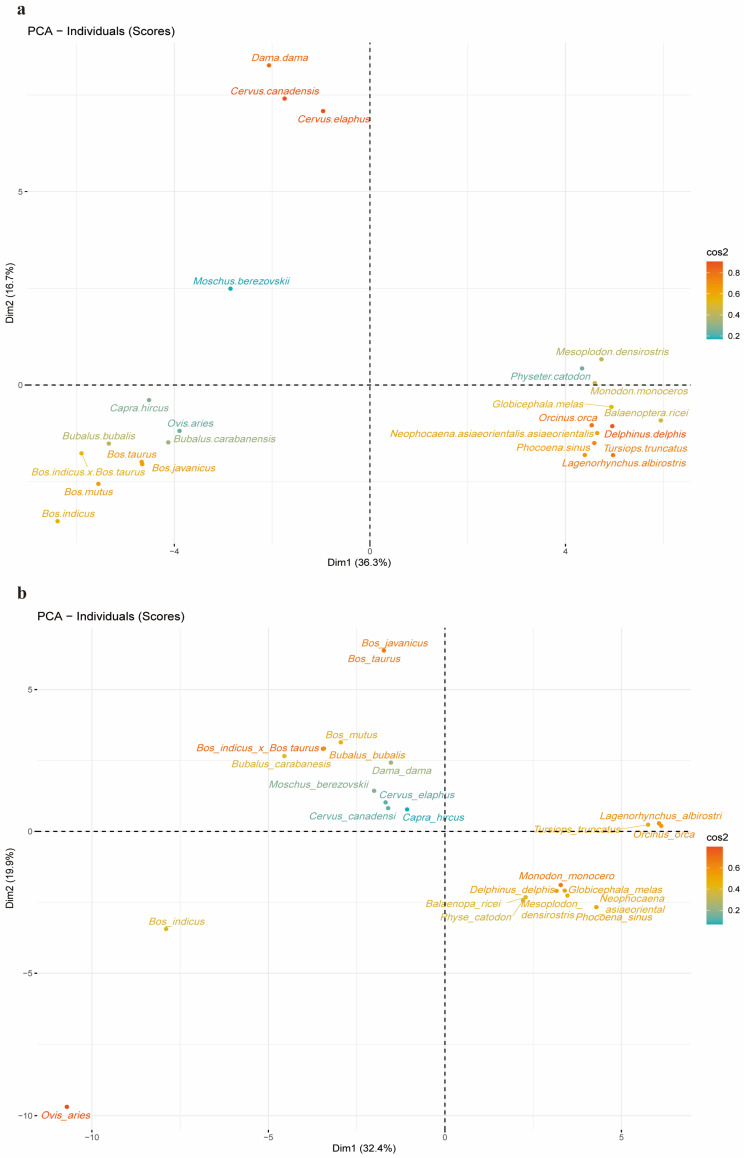
Species dimensionality reduction cluster analysis. (**a**) Construction of species PCA dimensionality reduction cluster analysis based on the RSCU values of the CDS sequence codon of the *DDR1* gene. (**b**) Construction of species PCA dimensionality reduction cluster analysis based on the RSCU values of the CDS sequence codon of the EHF TF.

To further investigate codon preference and species affinity, we performed a PCA for downclustering. Regarding the *DDR1* gene, the species *Ovis aries*, *Capra hircus*, *Bubalus bubalis*, *Bubalus carabanensis*, *Bos javanicus*, *Bos indicus* × *Bos taurus*, *Bos taurus*, *Bos mutus*, and *Bos indicus* formed a cluster (Figure 3a). In contrast, *Bos javanicus* and *Bos taurus* constituted a separate cluster and exhibited a closer relationship with the EHF TF (Figure 3b). *Moschus berezovskii*, *Dama dama, Cervus elaphus*, *Cervus canadensis*, *Bubalus carabanensis*, *Bubalus bubalis*, *Bos indicus* × *Bos taurus*, *Bos mutus*, and *Capra hircus* formed a cluster, although the related species *Bubalus carabanensis*, *Bubalus bubalis*, and *Bos mutus* exhibited similar affinities. Although dimensionality reduction revealed that certain codons contributed more to *DDR1* than others, the codons GUG, UGU, GUA, UUG, AGG, UGC, and CUA contributed more to *DDR1*. In contrast, codons ACG, AUA, CGC, UCG, ACA, GUU, CCU, AUC, and AUU contributed the most to the EHF TF (Figure S2). The results demonstrated consistency in the predicted phylogenetic relationships and CUB between *Bos javanicus* and *Bos taurus*, as well as among *Bubalus bubalis*, *Bubalus carabanensis*, and *Bos mutus* for the *DDR1* and EHF TFs. This finding provides further evidence to support the hypothesis that CUB is associated with phylogenetic relationships between species.

### 2.6. Correlation Analysis of Codon Usage Preference between DDR1 Gene and EHF

**Figure 4:**
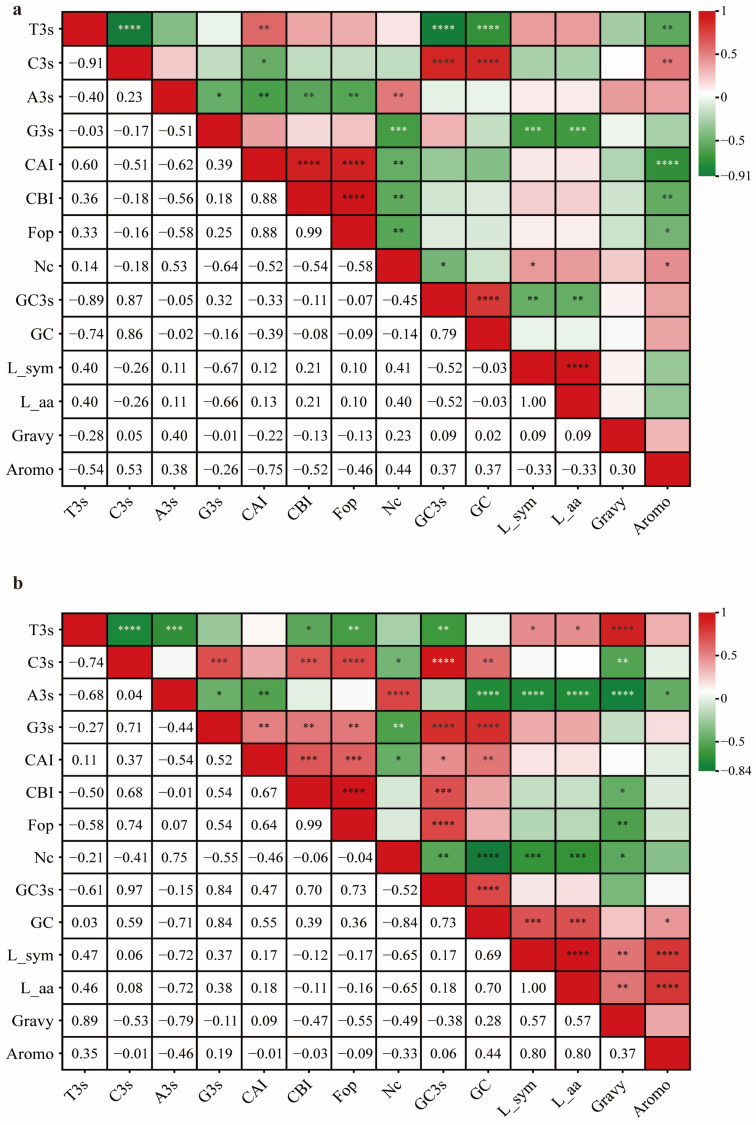
Correlation analysis of codon metrics (* for *p* ≤ 0.05; ** for *p* ≤ 0.01; *** for *p* ≤ 0.001; **** for *p* ≤ 0.0001). (**a**) Correlation analysis of codon metrics based on CDS sequences of *DDR1* gene, (**b**) correlation analysis of codon metrics based on CDS sequences of EHF TFs.

To investigate the factors that influence codon preference in *DRR1* and EHF, we conducted correlation analyses to examine the relationship between the 14 major contribution indices and their respective codons. Significant correlations were identified between the indices of ENC, CAI, and GC3s among the *DDR1* genes and EHF. The correlation coefficients between ENC and CAI were all significantly negative (*DDR1*: r = −0.52, *p* < 0.01; EHF: r = −0.46, *p* < 0.05) (Figure S3). This suggests that the codon usage of *DDR1* and EHF may have been influenced by gene selection and regulation, indicating that codon optimization may have been employed during evolution to enhance translation efficiency and gene expression levels. Furthermore, significant negative correlations were observed between the GC3s and ENC correlation coefficients (*DDR1* gene: r = −0.45, *p* < 0.05; EHF: r = −0.52, *p* < 0.01) (Figure 4a,b). It is plausible that a gene’s favored codon may have been influenced by the presence of GC3s, which optimized codon usage patterns during evolution to enhance gene expression and translation efficiency. This indicated a potential correlation between the GC content at the third position of the codon and the observed pattern of codon usage.

### 2.7. Analysis of DDR1 Gene and EHF Third Codon Bias

**Figure 5:**
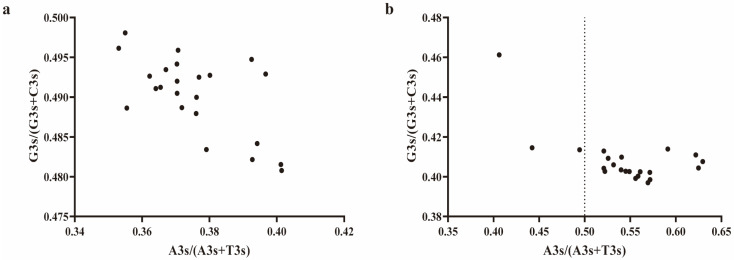
PR2 plot analysis. (**a**) Third codon preference analysis of the *DDR1* gene; (**b**) third codon preference analysis of the EHF TF. The dotted line represents the theoretical codon base usage frequency under mutation pressure, where A/T and C/G would be used at equal frequencies. The dots indicate the actual codon usage frequencies, reflecting the combined influences of both mutation pressure and natural selection.

To investigate codon usage patterns, selective pressures, and biological evolution, as well as gaining a deeper understanding of their functions and regulatory mechanisms, we conducted CDS codon parity rule 2 (PR2) analysis of the *DDR1* and EHF TFs. The values of [A3/(A3 + U3)] and [G3/(G3 + C3)] in the *DDR1* CDS codon were both lower than 0.5 (Figure 5a). In the case of EHF, the value of [A3/(A3 + U3)] was greater than 0.5, whereas the value of [G3/(G3 + C3)] was lower than 0.5 (Figure 5b). These findings suggest that a multitude of factors, including evolutionary pressures and selective forces, contribute to the overall complexity of codon usage patterns observed in the *DDR1* gene and EHF.

### 2.8. Multidimensional Clustering Analysis of CAI for DDR1–EHF Based on K-Means

**Figure 6:**
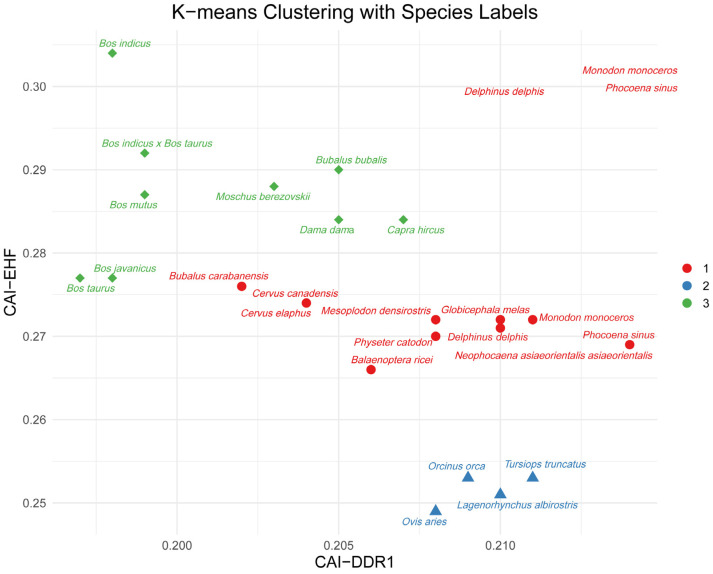
Multidimensional clustering analysis of CAI for *DDR1*–EHF based on k-means.

To further examine the genetic correlation between the *DDR1* and EHF codons, CAI was employed as a codon bias to assess the genes. Following CAI normalization of *DDR1* and EHF, the results demonstrated that *Bubalus bubalis*, *Bos taurus*, *Bos indicus* × *Bos taurus*, *Bos javanicus*, *Bos mutus*, *Dama dama*, *Capra hircus*, *Moschus berezovskii*, and *Bos indicus* formed one of three clusters (Figure 6). Bovine animals exhibited a closer genetic relationship, with *Bos taurus* displaying a closer affinity to *Bos javanicus* than to other species within the same group. The codon sequences of the *DDR1* gene and EHF TF exhibited notable similarities regarding species genetic linkages, as revealed through multidimensional prediction.

## 3. Discussion

Codon preference selection directly influences gene expression, and the use of optimal codons can significantly enhance translation efficiency and accuracy [48]. Therefore, changes in the GC content ratio affect the selection of codons and amino acids. Studies in mammals have shown that genes with a higher GC content often exhibit higher expression levels [49]. Additionally, TFs influence codon usage patterns, and significant correlations have been observed between the GC3 content and selected encoded amino acids [50,51]. This study analyzed the CDS sequences of *DDR1* and its predicted EHF TF in 24 randomly selected species, and the results show that the RSCU exhibited a preference for G/C-ending codons. Although the GC3 value of the EHF TF was lower than that of the *DDR1* gene, both still showed a preference for C and G at the third codon position. The study also revealed that the human genome exhibits a preference for G/C-ending codons [52]. For instance, *Daphnia* and *Drosophila melanogaster* have been shown to exhibit a preference for G/C, while *Plasmodium falciparum* has been found to exhibit a preference for A/T [53,54]. Although different species exhibit varying codon preferences for the same gene [55], the expression levels of *DDR1* and EHF TFs may still be correlated with the GC content in the 24 randomly selected mammalian species.

During biological evolution, CUB has been primarily influenced by the combined effects of mutation and selection pressure [56]. The impact of translational selection and mutational pressure on CUB is often illustrated through the correlation between ENC and CAI values [57,58,59]. If the correlation (r) between translational selection and mutation approaches equals −1, it indicates that translational selection exerts more influence than mutations. Conversely, if r is close to 0, it indicates no correlation, implying that mutations might have a greater impact than translational selection [60]. The ENC-GC3s plot is typically used to determine CUB [61]. We conducted a correlation analysis of codon usage in the CDS regions of *DDR1* and EHF, and the results indicate that codon usage is more influenced by gene selection and regulation. Furthermore, the data suggest that the preference for codons is influenced by the combined action of mutational pressure and selection forces. The optimal codons for *DDR1* and EHF were AGG, CCC, and GGA. The negative correlation between CG3 and ENC, approaching r = −0.5 (*DDR1* gene: r = −0.52; EHF: r = −0.46), indicated the joint action of selection and mutation pressures, which is consistent with the reduced number of optimal codons. In mammals, mutational pressures or selection forces are believed to significantly affect CUB [62,63]. The differences in the results between *DDR1* and EHF may suggest that both have maintained a high degree of conservation.

Numerous studies have demonstrated the link between codon usage and species phylogenetic relationships [64,65]. Previous research has also shown that both genes and TFs exhibit codon preferences [43,66]. This study jointly analyzed the codon bias of the *DDR1* gene and predicted the codon preferences of the EHF TF. Through phylogenetic tree analysis, hierarchical clustering, PCA, and k-means clustering, it was revealed that the codon usage frequency in *DDR1* and EHF was similar among species with close phylogenetic relationships. This was further confirmed in the k-means clustering analysis after CAI homogenization of *DDR1* and EHF, where *Bos javanicus* and *Bos taurus* exhibited a closer phylogenetic relationship. Additionally, aside from the 0.001 and 0.18 CAI and ENC differences in *DDR1*, respectively, both *DDR1* and EHF showed significant similarities in codon parameter values. This indicates a significant correlation between species phylogeny and the codon preferences of the *DDR1* gene and EHF TF, potentially providing a new perspective for evolutionary analysis of species relationships.

Previous studies have shown that genes use codons to encode amino acid sequences, thereby defining the translation rules from nucleic acids to proteins [67,68,69]. TFs bind to specific DNA sequences to regulate transcription, linking gene regulation with signal transduction [70,71]. Codon optimization is a valuable method for precisely regulating gene expression [27]. Given that dairy production is an important economic trait in global livestock farming, examining the CUB of *DDR1* and EHF TFs can influence the expression of codons related to dairy production genes through gene editing techniques. This approach could enhance the roles of *DDR1* and EHF in dairy production and lead to improvements in breeding strategies. Research on CUB plays a crucial role in regulating gene expression and protein function; however, this field has not been fully explored with respect to cattle. Therefore, this study is the first to reveal significant characteristics of codon usage in *DDR1* and EHF, thus providing new research directions and enabling further investigation of codon preferences, ultimately helping increase the protein yield of genes and TFs [72,73]. Preliminary studies on codons in lactation-related genes and transcription factors offer a new theoretical pathway for improving milk production in cattle. Additionally, these studies elucidate the roles of codons in TFs, genes, and species phylogeny, broadening the prospects for cross-species comparative analyses, with potential applications in the genetic improvement of other economically important species. Future research could explore how adjusting codon usage patterns in key genes may enhance production performance and biological adaptability, thereby delivering greater economic benefits to livestock farming.

## 4. Materials and Methods

### 4.1. DDR1 Gene Data and EHF TF Data Collection

Aquatic and terrestrial animals were randomly selected to represent a specific number of species in each genus. This was done to increase the accuracy of the results and minimize the deliberate nature of the screening process. Twenty-four species carrying *DDR1* were randomly selected for screening. For further details, refer to Table S4. The CDS format of the *DDR1* gene for all species was obtained from the National Center for Biotechnology Information (NCBI) GenBank database (http://www.ncbi.nlm.nih.gov/, accessed on 15 January 2024).

The Animal TFDB 4.0 (https://guolab.wchscu.cn/AnimalTFDB4/, accessed on 15 January 2024) was used to annotate and predict the animal TFs [11]. The database then predicted the corresponding TFs based on the *DDR1* promoter sequences, resulting in the highest ranked TF, EHF. Upon completion of the aforementioned steps, the FASTA format containing the corresponding EHF for the 24 species was obtained from the NCBI GenBank.

### 4.2. Phylogenetic Trees and Hierarchical Cluster Analysis

The CodonW 1.4.4 software (CodonCode Corporation, Boston, MA, USA) was employed to analyze the *DDR1* gene and EHF CDS sequences and compute several metrics pertaining to CUB. The T3s, C3s, A3s, G3s, CAI, CBI, Fop, ENC, GC3s, GC, L_sym, L_aa, Gravy, and Aromo values were individually tabulated and summarized in Excel. A phylogenetic tree was constructed for the *DDR1* gene and EHF TF using the default elaboration of MEGA 7.0.12 (MEGA Limited, Dhaka, Bangladesh) and bootstrap values of 1000 replicates using the neighbor-joining (NJ) method. NJ is a distance-based method used to construct evolutionary trees from a pairwise evolutionary distance matrix of the given sequences, and it was applied to investigate the relationships between genes and species [74]. Furthermore, Chiplot (https://www.chiplot.online/, accessed on 16 January 2024) was used to generate correlation indicator plots and hierarchical cluster analyses for the 24 species, using codon and RSCU values, respectively.

### 4.3. Parametric Statistical Methods for Codons

In accordance with the findings from CodonW, the RSCU and FOC tables for the *DDR1* gene and EHF were constructed in Excel. The RSCU table, which represents the ratio of the observed frequency of a specific synonymous codon to its expected frequency under the assumption of random usage, was used to identify alterations in the usage patterns of all synonymous codons across the gene. A codon was considered used with a relatively high frequency if its RSCU value exceeded one. Codon correlation analysis is a common method for measuring preference [67]. The relative codon usage frequency intersection was determined using the online program Venny 2.1.0 (https://bioinfogp.cnb.csic.es/tools/venny/index.html, accessed on 16 January 2024).

The RSCU index was calculated using the following formula:

$$RSCU=\frac{G_{ij}}{\sum_{ji}^{N_{i}}G_{ij}}N_{i}$$

where $G_{ij}$ represents the number of observations for the *i*th codon of the *j*th amino acid, which contains a total of $N_{i}$ synonymous codons [55].

The expression levels of *DDR1* in the 24 species were ranked by size using the ENC values. The top five species were classified as having low expression, whereas the bottom five were considered to have high expression. The high-expression group comprised *Bos taurus*, *Bos indicus* × *Bos taurus*, *Bos javanicus*, *Bos indicus*, and *Bos mutus*, whereas the low-expression group comprised *Neophocaena asiaeorientalis*, *Balaenoptera ricei*, *Monodon monoceros*, *Orcinus orca*, and *Physeter catodon*. Subsequently, the ENCs and RSCUs of the selected species were matched and the mean RSCU values for the two sets of codons were determined independently. Subsequently, the difference between the RSCUs of the high and low groups was calculated to determine ΔRSCU. Zhang et al. conducted subsequent analyses after employing the same procedure for TFs involved in EHF [75].

The calculation formula is as follows:

$$∆RSCU={RSCU}_{highexpressions}-{RSCU}_{lowexpressions}$$

### 4.4. Codon Correlation and Third Codon Analysis

PR2 plot analysis and ENC and CAI correlation analyses of *DDR1* and EHF were performed using GraphPad Prism 10. ENC is a measure of the degree of random variability in codon usage, whereas CAI quantifies the relative fitness of specific codons [39,40]. The relationship between translational selection and mutational pressure on CUB is frequently demonstrated using the fit between the ENC and CAI [57,58,59]. If the correlation coefficient (r) between translation selection and mutation is close to −1, then translation selection is more influential than mutation selection. Conversely, if r is near 0, then the two are not correlated and mutation may be more influential than translation selection [60]. Pass PR2 plots are frequently examined to investigate the impact of selection and mutational forces on codon usage. The effects of codon usage in response to selection pressures and mutations were frequently examined using pass PR2 plot analyses. The degree and direction of PR2 preference are represented by vectors originating from the center. The horizontal coordinate was [A3/(A3 + U3)], whereas the vertical coordinate was [G3/(G3 + U3)]. Both coordinates were centered at (0.5, 0.5) [76,77].

### 4.5. PCA and K-Means Cluster Analyses

The R software version 4.3.2 (R Foundation for Statistical Computing, Vienna, Austria) and the three R packages ggplot2, factoextra, and ggrepel were employed to plot the PCAs. For the *DDR1* gene and EHF, the downscaling and visualization of RSCU values and species data enabled the visual depiction of codon contributions and cluster analyses of genes and species contained in TFs [78,79].

The partitioning of the provided data points into k-prespecified, non-overlapping clusters, with each data point assigned to a single cluster, is referred to as k-means clustering [80]. The CAIs corresponding to the *DDR1* gene and the EHF TF of the 24 species were processed using the ggplot2 and ggrepel packages in the R software, version 4.3.2. The k-means clustering method was employed to analyze species clustering.

## 5. Conclusions

This study is the first to simultaneously analyze the CUB of the TF EHF and the *DDR1* gene associated with mammary traits. The results demonstrate that both the *DDR1* gene and the predicted EHF TF exhibit a preference for codons ending in C and G. Phylogenetic tree construction, hierarchical clustering, PCA, and k-means clustering revealed that CUB is closely tied to phylogenetic relationships among species. Species with similar codon usage patterns tend to share closer evolutionary relationships. Furthermore, the codon usage patterns of EHF and *DDR1* are shaped by both selective pressures and mutational forces. Despite some differences in codon usage among species, phylogenetically related species tend to display similar codon preferences.

These findings suggest that EHF may function as a key TF regulating *DDR1* expression, particularly in the context of genetic regulation of milk production. This study offers new theoretical insights into improving mammary traits and advancing cattle breeding strategies. Additionally, it opens new research avenues for breeding improvements in other economically important species.

## Figures and Tables

**Table 1:** Coding sequence features of the *DDR1* gene in 24 species.

Species	T3s/%	C3s/%	A3s/%	G3s/%	CAI	CBI	Fop	Nc	GC3s/%	GC/%	Gravy	Aromo
***Bos taurus***	17.23	47.39	11.21	44.48	0.197	0.071	0.442	41.49	75.90	63.30	−0.23	0.09
***Bos indicus* × *Bos taurus***	17.49	46.58	11.30	45.61	0.199	0.066	0.441	41.59	75.70	62.90	−0.22	0.09
***Bos javanicus***	17.23	47.39	11.21	44.48	0.198	0.072	0.443	41.67	75.90	63.30	−0.22	0.09
***Bos indicus***	16.73	47.94	11.22	44.39	0.198	0.082	0.448	41.72	76.30	63.40	−0.20	0.09
***Bos mutus***	17.04	47.72	11.02	44.43	0.199	0.082	0.449	41.78	76.20	63.30	−0.20	0.09
***Bubalus carabanensis***	16.82	47.85	11.27	44.44	0.202	0.083	0.449	41.80	76.20	63.40	−0.22	0.09
***Bubalus bubalis***	17.08	47.13	11.23	45.81	0.205	0.081	0.450	41.82	76.10	63.00	−0.21	0.09
***Capra hircus***	17.21	47.40	10.41	46.00	0.207	0.087	0.453	41.85	76.60	63.30	−0.22	0.09
***Ovis aries***	17.98	46.59	10.59	45.8	0.208	0.088	0.454	41.94	75.80	63.00	−0.21	0.09
***Cervus elaphus***	18.77	46.28	11.11	44.23	0.204	0.083	0.450	41.96	74.60	62.90	−0.22	0.09
***Dama dama***	18.41	46.87	11.24	43.86	0.205	0.087	0.452	42.00	74.90	63.00	−0.22	0.09
***Moschus berezovskii***	18.12	46.68	10.92	44.48	0.203	0.074	0.444	42.00	75.30	63.20	−0.21	0.09
***Cervus canadensis***	18.90	46.15	10.82	44.53	0.204	0.083	0.450	42.01	74.70	63.00	−0.22	0.09
***Globicephala melas***	18.35	47.23	10.12	45.13	0.210	0.094	0.456	42.02	75.90	63.20	−0.27	0.09
***Phocoena sinus***	18.28	46.74	10.53	45.13	0.214	0.101	0.460	42.02	75.60	63.20	−0.22	0.09
***Mesoplodon densirostris***	18.54	46.08	11.37	44.76	0.208	0.083	0.449	42.14	74.70	62.90	−0.22	0.09
***Lagenorhynchus albirostri***	18.12	46.68	10.66	45.21	0.210	0.097	0.457	42.16	75.60	63.10	−0.22	0.09
***Delphinus delphis***	18.38	46.41	10.66	45.21	0.210	0.091	0.454	42.19	75.40	63.00	−0.22	0.09
***Tursiops truncatus***	18.12	46.81	10.66	45.06	0.211	0.095	0.456	42.19	75.60	63.10	−0.22	0.09
***Neophocaena asiaeoriental asiaeoriental***	18.54	46.48	10.53	45.13	0.214	0.101	0.460	42.21	75.30	63.10	−0.22	0.09
***Balaenoptera ricei***	19.35	45.75	10.56	45.05	0.206	0.076	0.445	42.32	74.60	62.80	−0.23	0.09
***Monodon monoceros***	18.64	46.28	11.24	44.46	0.211	0.096	0.456	42.52	74.70	62.80	−0.22	0.09
***Orcinus orca***	18.38	46.28	10.81	45.21	0.209	0.090	0.452	42.57	75.30	63.00	−0.22	0.09
***Physeter catodon***	19.17	45.63	10.55	45.28	0.208	0.089	0.452	42.66	74.80	63.00	−0.22	0.09

**Table 2:** Coding sequence signatures of EHF in 24 species.

Species	T3s/%	C3s/%	A3s/%	G3s/%	CAI	CBI	Fop	Nc	GC3s/%	GC%	Gravy	Aromo
***Bos taurus***	21.38	46.54	30.92	32.87	0.277	0.215	0.554	57.52	59.90	49.10	−0.95	0.10
***Bos indicus* × *Bos taurus***	22.67	49.78	25.73	34.02	0.292	0.226	0.56	54.01	63.10	50.70	−0.81	0.11
***Bos javanicus***	21.38	46.54	30.92	32.87	0.277	0.215	0.554	57.52	59.90	49.10	−0.95	0.10
***Bos indicus***	27.37	47.37	21.71	33.54	0.304	0.187	0.536	50.46	61.70	50.80	−0.47	0.10
***Bos mutus***	21.95	49.76	28.04	33.52	0.287	0.206	0.55	53.46	62.30	50.40	−0.87	0.12
***Bubalus carabanensis***	25.57	47.03	25.00	33.16	0.276	0.199	0.542	54.68	60.90	50.00	−0.64	0.12
***Bubalus bubalis***	24.22	48.88	26.34	33.16	0.29	0.204	0.548	54.06	61.60	50.10	−0.79	0.11
***Capra hircus***	23.21	48.66	27.80	32.81	0.284	0.218	0.555	55.03	61.20	50.20	−0.80	0.11
***Ovis aries***	34.60	37.26	23.67	31.90	0.249	0.108	0.483	54.81	53.60	49.40	−0.28	0.12
***Cervus elaphus***	23.21	49.11	25.73	34.02	0.274	0.187	0.537	53.93	62.60	50.80	−0.79	0.11
***Dama dama***	22.64	50.00	24.74	33.71	0.284	0.242	0.567	53.56	63.60	51.00	−0.68	0.12
***Moschus berezovskii***	21.88	50.00	25.73	34.72	0.288	0.23	0.562	53.42	63.70	51.20	−0.78	0.11
***Cervus canadensis***	23.21	49.11	25.24	34.54	0.274	0.187	0.537	54.31	63.00	50.90	−0.79	0.11
***Globicephala melas***	21.17	50.45	26.79	33.67	0.272	0.198	0.544	52.53	63.30	50.90	−0.77	0.11
***Phocoena sinus***	21.17	49.55	28.23	33.33	0.269	0.185	0.537	53.24	62.30	50.60	−0.76	0.11
***Mesoplodon densirostris***	22.97	49.55	26.96	33.51	0.272	0.175	0.532	52.32	62.10	50.20	−0.80	0.12
***Lagenorhynchus albirostri***	19.23	48.08	32.03	32.64	0.251	0.175	0.532	56.16	60.70	49.10	−0.92	0.10
***Delphinus delphis***	21.72	50.23	26.44	33.85	0.271	0.202	0.546	53.08	63.20	50.70	−0.78	0.11
***Tursiops truncatus***	19.87	46.79	32.68	32.64	0.253	0.175	0.532	57.44	59.70	48.80	−0.92	0.10
***Neophocaena asiaeoriental asiaeoriental***	21.17	49.55	28.23	33.33	0.269	0.185	0.537	53.24	62.30	50.60	−0.76	0.11
***Balaenoptera ricei***	20.27	51.35	27.05	34.02	0.266	0.198	0.544	53.15	64.10	50.80	−0.80	0.11
***Monodon monoceros***	20.72	50.90	27.40	33.51	0.272	0.199	0.544	53.05	63.30	50.70	−0.76	0.11
***Orcinus orca***	19.23	47.44	32.68	32.64	0.253	0.175	0.532	56.58	60.20	49.00	−0.92	0.10
***Physeter catodon***	21.62	50.45	27.05	33.51	0.27	0.198	0.544	52.62	63.00	50.60	−0.79	0.11

T3s: frequency of the nucleotide T at the third codon position; C3s: frequency of the nucleotide C at the third codon position; A3s: frequency of the nucleotide A at the third codon position; G3s: frequency of the nucleotide G at the third codon position; CAI: codon adaptation index; CBI: codon bias index; Fop: frequency of optimal codons; Nc: effective number of codons; GC3s: frequency of the nucleotides G + C at the third codon position; GC: G + C content; Gravy: hydrophilicity index of the protein; Aromo: proportion of aromatic amino acids in the protein.

**Table 3:** Relative synonymous codon usage values and number of codons for 24 *DDR1* genes.

Amino Acid	Codon	Number	RSCU	Amino Acid	Codon	Number	RSCU
Phe	UUU	318	0.77	Ala	GCU	253	0.60
	UUC *	506	1.23		GCC *	1005	2.38
Leu	UUA	105	0.24		GCA	177	0.42
	UUG	155	0.36		GCG	252	0.60
	CUU	182	0.42	Tyr	UAU	281	0.87
	CUC *	664	1.52		UAC *	366	1.13
	CUA	141	0.33	His	CAU	150	0.58
	CUG *	1368	3.14		CAC *	369	1.42
Ile	AUU	74	0.36	Gln	CAA	74	0.19
	AUC *	522	2.52		CAG *	682	1.81
	AUA	26	0.13	Asn	AAU	212	0.66
Val	GUU	118	0.32		AAC *	427	1.34
	GUC	301	0.83	Lys	AAA	45	0.16
	GUA	75	0.21		AAG *	513	1.84
	GUG *	958	2.63	Asp	GAU	475	0.79
Ser	UCU	157	0.75		GAC *	726	1.21
	UCC *	289	1.38	Glu	GAA	150	0.25
	UCA	77	0.37		GAG *	1049	1.75
	UCG	79	0.38	Cys	UGU	127	0.62
	AGU	148	0.71		UGC *	282	1.38
	AGC *	505	2.41	Arg	CGU	95	0.35
Pro	CCU	423	0.89		CGC *	428	1.59
	CCC *	864	1.82		CGA	127	0.47
	CCA	343	0.72		CGG *	630	2.33
	CCG	268	0.56		AGA	62	0.23
Thr	ACU	124	0.60		AGG *	277	1.03
	ACC *	443	2.15	Gly	GGU	151	0.30
	ACA	126	0.61		GGC *	825	1.64
	ACG	133	0.65		GGA	268	0.53
					GGG *	767	1.53

“*” Indicates more frequently used codons (RSCU > 1).

**Table 4:** Relative synonymous codon usage values and number of codons for 24 EHF.

Amino Acid	Codon	Number	RSCU	Amino Acid	Codon	Number	RSCU
Phe	UUU	87	0.64	Ala	GCU	37	0.54
	UUC *	164	1.36		GCC *	116	1.76
Leu	UUA	71	0.70		GCA	63	0.97
	UUG	74	0.73		GCG	49	0.72
	CUU	66	0.61	Tyr	UAU	110	0.83
	CUC *	187	1.74		UAC *	156	1.17
	CUA	33	0.32	His	CAU	81	0.63
	CUG *	201	1.91		CAC *	165	1.37
Ile	AUU	85	0.89	Gln	CAA	67	0.35
	AUC *	197	2.07		CAG *	327	1.65
	AUA	3	0.03	Asn	AAU	189	0.85
Val	GUU	23	0.36		AAC *	253	1.15
	GUC *	56	1.08	Lys	AAA *	242	1.06
	GUA *	67	1.27		AAG	214	0.94
	GUG *	69	1.29	Asp	GAU	73	0.46
Ser	UCU	32	0.36		GAC *	243	1.54
	UCC *	109	1.24	Glu	GAA *	276	1.24
	UCA	35	0.41		GAG	168	0.76
	UCG	10	0.12	Cys	UGU	3	0.04
	AGU	81	0.91		UGC *	107	1.96
	AGC*	259	2.95	Arg	CGU	34	0.62
Pro	CCU*	95	1.35		CGC	33	0.59
	CCC	66	0.96		CGA *	61	1.10
	CCA*	64	1.00		CGG *	68	1.36
	CCG	45	0.69		AGA	77	1.47
Thr	ACU	74	0.66		AGG *	48	0.88
	ACC*	211	1.90	Gly	GGU	70	0.70
	ACA	110	1.00		GGC	101	0.99
	ACG	51	0.44		GGA	95	0.94
					GGG *	137	1.38

“*” indicates codons more frequently used codons (RSCU > 1).

## Data Availability

Data are contained within the article and Supplementary Materials.

## Supplementary Materials

The following supporting information can be downloaded from https://www.mdpi.com/article/10.3390/ijms251910696/s1.
