# Supplementary material for: Codon Bias of the DDR1 Gene and Transcription Factor EHF in Multiple Species
Source: Int J Mol Sci. 2024 Oct 4;25(19):10696. doi: 10.3390/ijms251910696 (PMC11477322; doi:10.3390/ijms251910696)
Supplement: Supplementary file 1 [file ijms-25-10696-s001.zip › supplementary.pdf]

Supplementary Materials: Codon bias of *DDR1* gene and transcription factor EHF in multispecies

Zhiyong Zhang †, Wenxi Li †, Ziyang Wang, Shuya Ma, Fangyuan Zheng, Hongyu Liu, Xiaodong Zhang, Yueyun Ding, Zongjun Yin, and Xianrui Zheng *

.

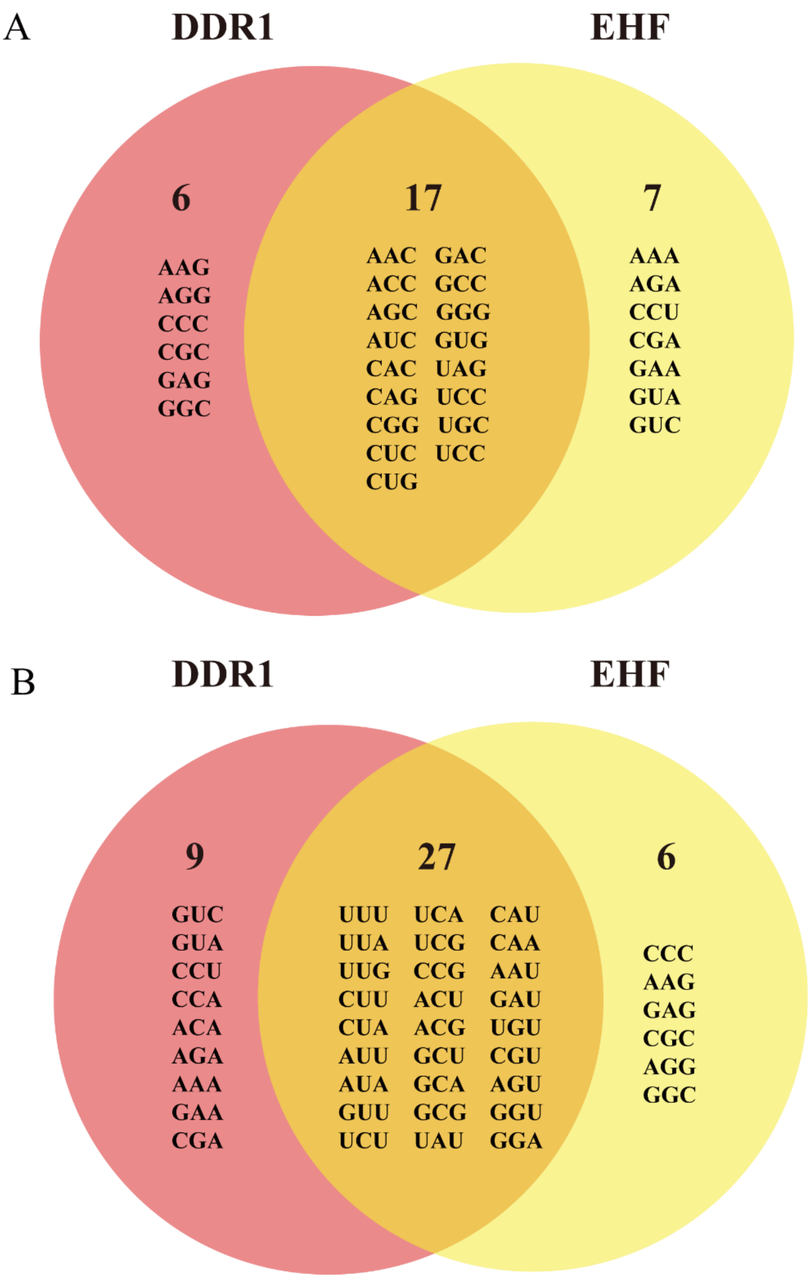

Figure S1. (**A**) Venn diagrams of codons (RSCU>1) in the CDS region of the *DDR1* gene and the EHF, (**B**) Venn diagrams of codons (RSCU<1) in the CDS region of the *DDR1* gene and the EHF.

A

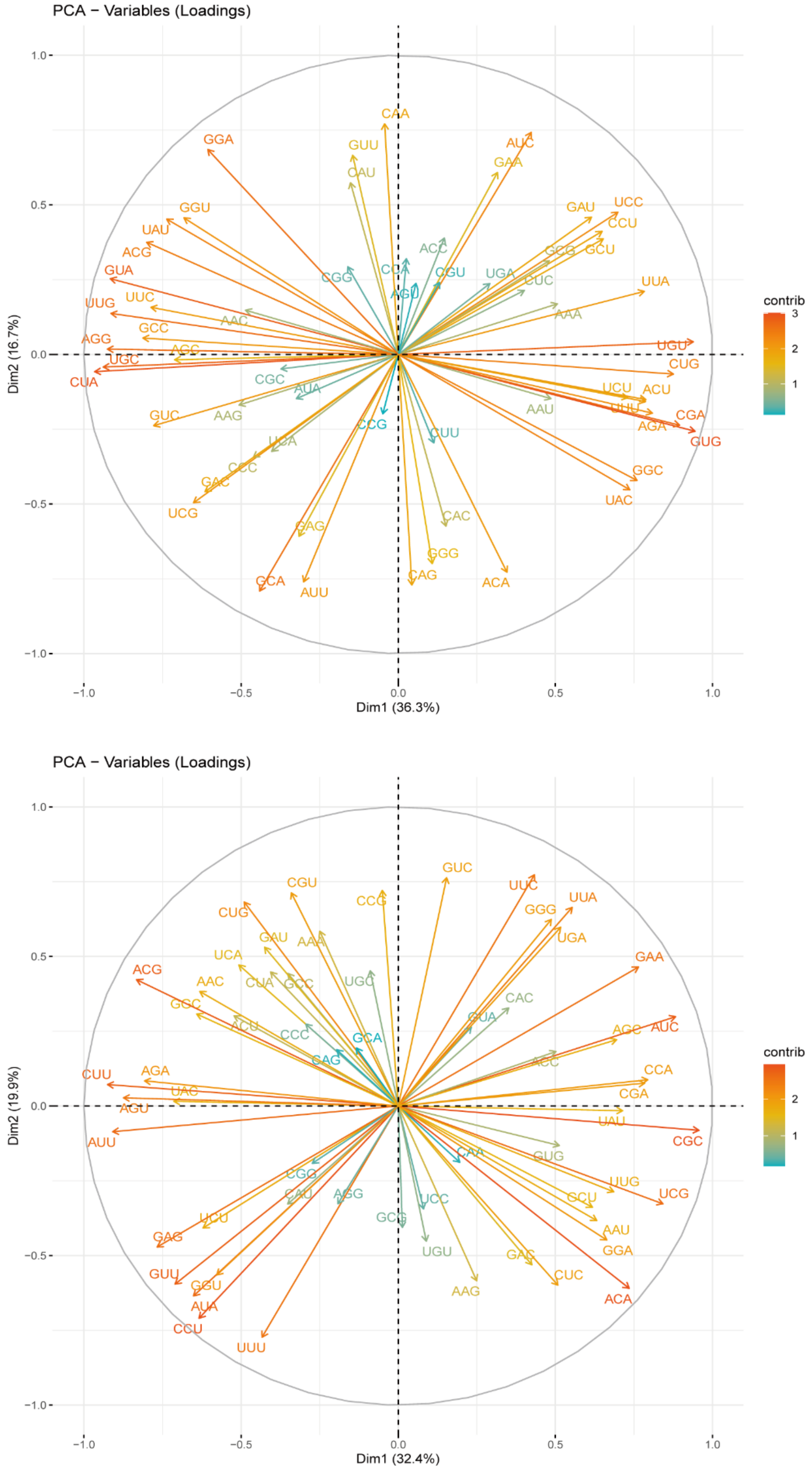

Figure S2. PCA dimensionality reduction clustering analysis of RSCUs corresponding to codons in 24 species. (**A**) PCA dimensionality reduction clustering analysis of the contribution of codons corresponding to the CDS region of the *DDR1* gene, (**B**) PCA dimensionality reduction clustering analysis of the contribution of codons corresponding to the CDS region of the EHF.

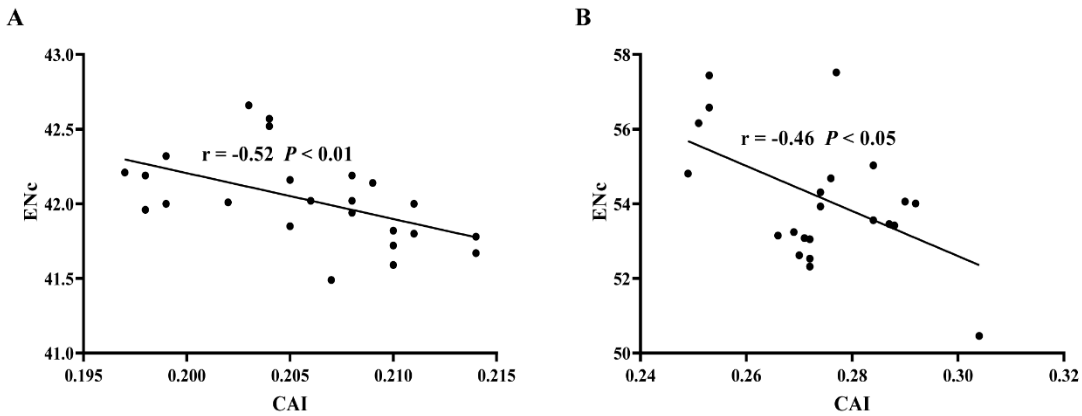

Figure S3. (**A**) ENc-CAI coanalysis of the *DDR1* gene, (**B**) ENc-CAI coanalysis of the EHF.

Table S1. RSCU in the high expression group, low expression group of *DDR1* gene; **Table S2.** RSCU in the high expression group, low expression group of EHF; **Table S3.** Accession number corresponding to the 24 species containing the *DDR1* gene

Please find Table S1–S3 in the supplementary material.
